# Automatic extraction of transcriptional regulatory interactions of bacteria from biomedical literature using a BERT-based approach

**DOI:** 10.1093/database/baae094

**Published:** 2024-08-30

**Authors:** Alfredo Varela-Vega, Ali-Berenice Posada-Reyes, Carlos-Francisco Méndez-Cruz

**Affiliations:** Programa de Genómica Computacional, Centro de Ciencias Genómicas, UNAM, Av. Universidad S/N Col. Chamilpa, Cuernavaca, Morelos 62210, México; Laboratorio de Microbiología, Inmunología y Salud Pública, Facultad de Estudios Superiores Cuautitlán, UNAM, Carretera Cuautitlán-Teoloyucan Km. 2.5, Xhala, Cuautitlán Izcalli, Estado de México 54714, México; Programa de Genómica Computacional, Centro de Ciencias Genómicas, UNAM, Av. Universidad S/N Col. Chamilpa, Cuernavaca, Morelos 62210, México

## Abstract

Transcriptional regulatory networks (TRNs) give a global view of the regulatory mechanisms of bacteria to respond to environmental signals. These networks are published in biological databases as a valuable resource for experimental and bioinformatics researchers. Despite the efforts to publish TRNs of diverse bacteria, many of them still lack one and many of the existing TRNs are incomplete. In addition, the manual extraction of information from biomedical literature (“literature curation”) has been the traditional way to extract these networks, despite this being demanding and time-consuming. Recently, language models based on pretrained transformers have been used to extract relevant knowledge from biomedical literature. Moreover, the benefit of fine-tuning a large pretrained model with new limited data for a specific task (“transfer learning”) opens roads to address new problems of biomedical information extraction. Here, to alleviate this lack of knowledge and assist literature curation, we present a new approach based on the Bidirectional Transformer for Language Understanding (BERT) architecture to classify transcriptional regulatory interactions of bacteria as a first step to extract TRNs from literature. The approach achieved a significant performance in a test dataset of sentences of *Escherichia coli* (F1-Score: 0.8685, Matthew’s correlation coefficient: 0.8163). The examination of model predictions revealed that the model learned different ways to express the regulatory interaction. The approach was evaluated to extract a TRN of *Salmonella* using 264 complete articles. The evaluation showed that the approach was able to accurately extract 82% of the network and that it was able to extract interactions absent in curation data. To the best of our knowledge, the present study is the first effort to obtain a BERT-based approach to extract this specific kind of interaction. This approach is a starting point to address the limitations of reconstructing TRNs of bacteria and diseases of biological interest.

**Database URL:** https://github.com/laigen-unam/BERT-trn-extraction.

## Introduction

Manual extraction of relevant information from biomedical literature, named “literature curation,” has been the traditional way to extract, integrate, and organize knowledge in biological databases [1]. As manual curation is demanding and time-consuming, machine learning approaches of information extraction have been proposed for decades to support this task [2, 3]. Recently, language models based on pretrained transformers have gained interest as they have demonstrated surpassing the performance of classical machine learning approaches [4, 5]. Moreover, the benefit of fine-tuning a large pretrained model with new limited data for a specific task (“transfer learning”) opens roads to address new problems of information extraction [6].

A relevant problem is the extraction of biological networks from literature, which has been a topic of interest since a while ago [7]. Examples of these networks are transcriptional regulatory networks (TRNs) of bacteria, which are valuable resources for organizing and integrating the knowledge of transcriptional regulation. Transcription is a primary process in cells, in which the RNA is synthesized from a DNA template by the RNA polymerase [8]. Despite the efforts to publish TRNs of diverse bacteria, such as *Escherichia coli* [9] or *Salmonella* [10], there is a lack of these networks, the existing ones are incomplete, or they are not open access [11].

Here, we defined a TRN as a set of transcriptional regulatory interactions between transcription factors (TFs) and regulated genes or regulated operons (group of genes). These interactions play a leading role in the rapid response of bacteria to environmental signals, such as changes in nutrient availability, and molecular stressors of the niche they colonize [8, 12]. The study of these interactions may have an impact on relevant problems, such as antimicrobial resistance [13, 14], biotechnological processes [15], and public health [16].

In a transcriptional regulatory interaction, if the regulatory effect is of the “activation” type, the TF will promote the expression of the regulated gene. If the effect is of the “repression” type, the expression of the regulated gene will be inhibited by blocking the activity of the RNA polymerase [16, 17]. A regulatory interaction is therefore formed by a TF, a regulated gene/operon, and an effect, which may be “activation,” “repression,” or simply “regulation,” when the effect exists, but the type is not reported.

Here, to alleviate this lack of knowledge and to assist the manual curation of TRNs, we present a new approach for the extraction and classification of transcriptional regulatory interactions from biomedical literature, which is the first stage to reconstruct a TRN. We compared six state-of-the-art architectures based on the Bidirectional Transformers for Language Understanding (BERT) to find a best model [6]. To the best of our knowledge, the present study is the first effort to obtain a BERT-based model to extract this specific kind of interaction. Many of the recent works and emerging standard benchmarks for biomedical relation extraction, such as BLUE, preferably address drug–drug and chemical–protein interactions [18–20]. The ability of BERT-based pretrained models to achieve high performance by fine-tuning with limited data has made it possible to address new types of interactions, such as SNP-phenotype [21] and genes–antibiotic resistance [22].

We employed techniques of “biomedical relation extraction,” a task of natural language processing (NLP) that aims at predicting from an input text whether two or more mentions of entities have some relation and, in some cases, the type of relation (i.e. cause, binding, induction) [2, 5, 23]. This task has been defined as a classification task, where a set of sentences are classified in a category [24]. A common strategy is to predict whether an interaction is true or false (binary classification) given a pair of mentions of entities and a sentence.

In our approach, we classified the regulatory interaction in four categories (classes) depending on the type of effect of the TF that is expressed over the regulated element: “activator,” “repressor,” “regulator,” and “no_relation” if there is no effect. Thus, our problem is defined as a multiclass classification task with exclusive categories. Examples of sentences of the three regulatory effects are shown in Table 1. Note that the way to express each type of effect is not necessarily done with verbs like “activate,” “repress,” or “regulate.” Furthermore, the way to syntactically express the interaction is not in active form (“[Transcription Factor] activates [Gene]”). This makes our problem an interesting challenge for BERT-based architectures.

**Table 1.** Examples of sentences expressing regulatory interactions between TFs and regulated elements. Entity mentions in boldface

Category	TF	Regulated element	Sentence
Activator	CadC	*cadBA*	“**CadC**-mediated activation of the ***cadBA*** promoter in *Escherichia coli*.” [52]
Repressor	MarA	*purA*	“In the intact cell, transcription of ***purA*** was decreased in cells constitutively producing **MarA**.” [53]
Regulator	Fnr	*dmsA*	“Thus, the ***dmsA*** promoter exhibits a preference for −41.5 target sites like other **Fnr**-regulated class II promoters.” [54]

We used a dataset of 1562 sentences of *E. coli* K-12 manually labeled in one of the four categories. This was randomly split in train (999 sentences), dev (250 sentences), and test (313 sentences) datasets to fine-tune and evaluate six state-of-the-art BERT-based architectures: BERT [6], BioBERT [24], BioLinkBERT [25], BioMegatron [26], BioRoBERTa [27], and LUKE [28]. Finally, the best model was applied for the extraction of a TRN of *Salmonella enterica* serovar Typhimurium using 264 complete articles. We evaluated the extraction utilizing a dataset of 3005 sentences with regulatory interactions extracted from the same articles by manual curation. Our findings demonstrated that our model may be used to extract regulatory interactions from literature of diverse bacteria to assist manual curation.

## Materials and methods

**Figure 1.**
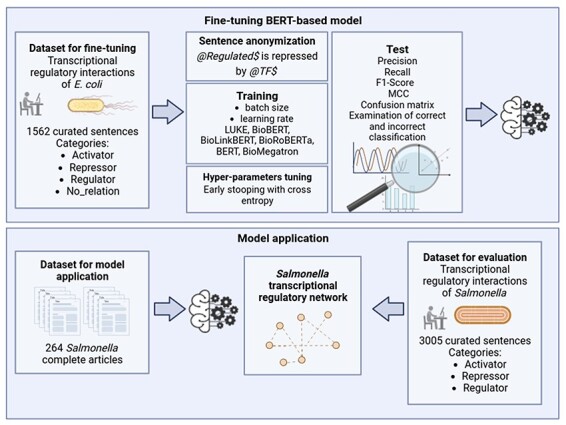
General scheme of the study. Created with BioRender.com.

A general graphical scheme of the study carried out in the present work is shown in Fig. 1 and is explained in detail in the subsequent sections.

### Dataset for fine-tuning

RegulonDB is the database with the main TRN publicly available for *E. coli* [9]. It comprises a large collection of regulatory interactions manually curated for decades from biomedical literature. Some time ago, the curation team of RegulonDB compiled a set of 1083 sentences containing regulatory interaction using an assisted-curation strategy [29]. This dataset was provided for our study by the RegulonDB curation team.

A limitation of this initial dataset was that mentions of TFs and regulated elements (genes and operons) within the sentences were not textually marked. In other words, we were given only the name of the TF, the name of the gene, the sentence, and the category of the regulatory effect. For example, in Table 2, we show the example of a sentence from the initial data set with one mention of a TF and two mentions of a regulated element, but the combination of mentions of entities (TF–gene) that actually express the interaction were not marked textually within the sentence by the RegulonDB team. This is important because to fine-tune a BERT-based pretrained model, it is necessary to indicate which mentions of the pair of entities (“target entities”) interact [18, 23, 24, 28]. Therefore, we perform the following tasks to curate the initial dataset of 1083 sentences to obtain a dataset suitable for fine-tuning a BERT-based pretrained model. Sentences with multiple mentions of both entities were duplicated as many times as pairs of entity mentions and then manually reviewed to select the sentence with the combination of the entity mentions expressing the interaction (see row 1 in Table 2). The remaining duplicated sentences were labeled with the category “no_relation,” as the combination of the entity mentions did not express the interaction (see row 2 in Table 2). The category “no_relation” has been previously proposed for a model to improve its performance by learning from negative examples [30].

**Table 2.** Example of a sentence with two combinations of entity mentions: one TF and two operons. Entity mentions in boldface. The combination of the entity mentions in sentence 1 expresses the interaction, whereas the combination of the entity mentions in sentence 2 does not. The sentence was recovered from [55]

#	Category	Sentence
1	*regulator*	“**RhaR** regulates transcription of ***rhaSR*** by binding promoter DNA spanning 32 to 82 relative to the *rhaSR* transcription start site”
2	*no_relation*	“**RhaR** regulates transcription of *rhaSR* by binding promoter DNA spanning 32 to 82 relative to the ***rhaSR*** transcription start site”

**Figure 2.**
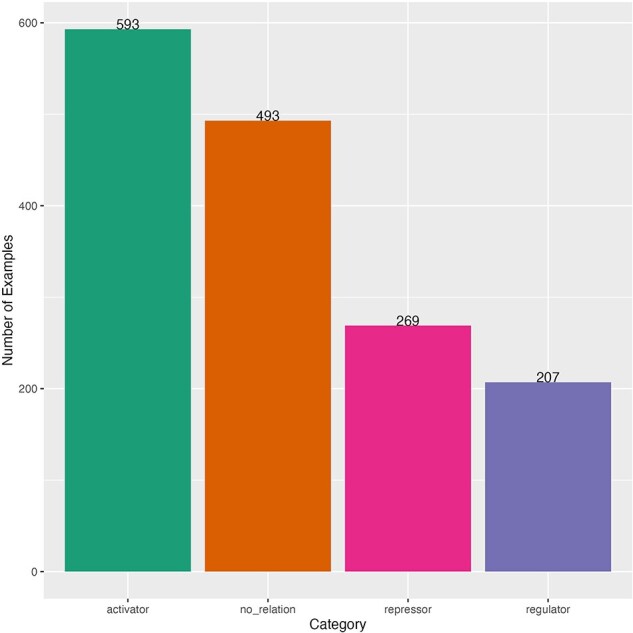
Distribution of curated sentences by category of the dataset for fine-tuning. Sentences from literature of *E. coli*.

After such curation, we obtained a dataset with 1562 sentences for fine-tuning. This dataset had an imbalanced distribution of sentences by category (Fig. 2). The majority category was “activator” (593 sentences) and the minority was “regulator” (207 sentences). The distribution of the length of the sentences (number of characters) was obtained to observe the heterogeneity and complexity of the dataset. The minimum sentence length was 27 characters and the maximum length was 665 characters (mean of 236 and median of 223) (Supplementary Fig. S1). Sentences come from 119 different scientific articles (Supplementary Fig. S2). Our dataset was randomly split in 80% for train-dev to find the best model and 20% for testing the best model (held-out dataset), then we split the train-dev dataset in 80% for training and 20% to find the best hyperparameters. The three datasets kept the same number of sentence distribution by category as the complete dataset (Supplementary Table S1).

### BERT-based architectures

The “Bidirectional Encoder Representations from Transformers” (BERT) is a state-of-the-art language model based on the transformer architecture. Its capability to generate complex language representations lies in the two principal mechanisms that it inherits from the Transformer Encoder, as well as the massive pretraining over two subsets of text (corpus) [6]. These two main mechanisms are the multihead self-attention and the position-wise fully connected feed-forward network, which, when assembled, can create multiple representations of a word and its context within a sentence.

The multihead self-attention measures similarity scores between each word in the vocabulary by using positional encoding and word vector representations to predict the likelihood of the next/previous occurring word or next sentence [31]. Meanwhile, the second mechanism captures textual patterns in the training examples, with each value inducing a distribution over the output vocabulary that adds up to the next word or sentence prediction [32]. BERT was released to the public domain with the weights and biases learned during the pretraining phase using 800 million words from BookCorpus and 2500 million words from English Wikipedia. That was the turning point for more architectures to emerge using a transfer learning approach [33], taking BERT architecture and its previously acquired knowledge to fine-tune it for executing a specific language task, to excel in the understanding of a specialized domain, or simply to improve its overall performance.

In addition to the original BERT architecture, we fine-tuned models of five additional BERT-based architectures: BioBERT, BioLinkBERT, BioMegatron, BioRoBERTa, and LUKE. The first four are biomedical domain-specific architectures and the last one is task-specific. The “Bidirectional Encoder Representations from Transformers for Biomedical Text Mining” (BioBERT) is a domain-specific model pretrained on 4.5 billion words from PubMed abstracts and 13.5 billion words from PubMed Central full-text articles [24]. BioLinkBERT is a model that incorporates link knowledge by pretraining the architecture with hyperlinks between Wikipedia articles and citation links (references) from 21 GB of PubMed articles [25]. BioMegatron is an architecture designed to extend the number of trainable parameters of BERT beyond 345 million by implementing efficient model parallelism. Moreover, BioMegatron extends the pretraining corpus by adding 4.5 billion words from PubMed abstracts and 1.6 billion words of a CC0-licensed Commercial Use Collection of the PMC full-text corpus [26].

BioRoBERTa is the biomedical domain version of the “Robustly Optimized BERT Approach” (RoBERTa), which pointed out that BERT was undertrained and should be trained longer, using bigger batches, longer sequences, and dynamically changing the masking pattern during training. Furthermore, 76 GB of English news articles were added, 38 GB of open web text content, and 31 GB of a dataset matching the story-like style of Winograd schemas [34]. For the biological domain version, BioRoBERTa uses 2.68 million full-text papers from The Semantic Scholar Open Research Corpus [27]. The “Language Understanding with Knowledge-based Embeddings” (LUKE) adjusts the training following the RoBERTa architecture. It also implements a new pretraining task, predicting randomly masked words and entities in a large entity-annotated corpus retrieved from Wikipedia. Moreover, a slight change in the self-attention mechanism was done to compute attention scores based on the type of token, creating the entity-aware self-attention mechanism [28].

### Experimental design

To fine-tune BERT-based models for relation extraction tasks, previous works have proposed that the entity mentions within the sentence must be anonymized [18, 23, 24, 28, 35]. For example, Lee *et al*. [24] employed the anonymization procedure to extract gene–disease relations using the predefined tags @GENE$ and @DISEASE$ (e.g. “Serine at position 986 of @GENE$ may be an independent genetic predictor of angiographic @DISEASE$”). Therefore, in each of the 1562 sentences, the mentions of the TF and the regulated element were anonymized using the @TF$ and @Regulated$ pre-defined tags, respectively (e.g. “@Regulated$ expression is repressed by @TF$”). The reason to anonymize the entity mentions is that this anonymization process allows the model to learn patterns associated with the relation between any TF and any regulated element. The model learns to predict the output category of the sentence using a distributional contextual representation of every word in the sentence. If we did not perform the anonymization process, the model may learn patterns associated to the specific name of the entities, so the model may fail when it was applied to a new dataset with different entity names.

The sentences with anonymized entities were input to the tokenizer of each architecture, which generated a numerical representation of each word so that the pretrained model could be fine-tuned. For the LUKE architecture, the start and the end positions of the pair of entity mentions within the sentence (”spans”) are required by its tokenizer, which subsequently generates the entity masking. Then, the sentence and the entity spans were input to LUKE architecture. For all architectures, the category for each sentence (“activator,” “repressor,” “regulator,” and “no_relation”) was also input.

We observed that most of the previous studies that fine-tuned pretrained BERT models, such as BERT [6], BioLinkBERT [25], BioMegatron [26], and LUKE [28], did not use a cross-validation strategy. Instead, these studies employed a hyperparameter tuning strategy. On account of that, to find the best model, we used the grid-search approach for the batch size and the learning rate based on the early stopping when the loss function does not improve in two epochs (patience = 2) using the dev dataset, a strategy that has also been recommended previously [36]. This hyperparameter tuning strategy provided us with an effective way to test our model’s robustness without the extensive computational demands associated with the fine-tuning cross-validation of pretrained BERT-based models. For multiclass classification problems, a common way to calculate the loss function is by means of the cross-entropy function [37, 38]. This function takes the output from the softmax activation function (the probability that an example belongs to each category), and calculates a scalar value quantifying the difference (error) between the predicted probability distribution $p$ and the true categories $y$:


(1) $$H\left( {p,y} \right) = - \mathop \sum \limits_i^{} {y_i}\ log\,\left( {{p_i}} \right)\,\,\,\,\,\,\,\,\,\,where\,i\, \in \,\left[ {1,N} \right],\,$$

where $p$ is a vector of probabilities from the softmax function of size $N$, that is, the number of categories; and $y$ is usually a binary vector of size $N$, where 1 indicates the true category and 0 the false one. For this function, the worst value is $ + \infty $ and the best value is 0.

We used the AdamW optimizer for all experiments. To conduct a fair compassion of models, we use the same grid of values for the batch size and the learning rate in all experiments. We selected these values from those commonly recommended for fine-tuning in the original articles of the six BERT-based architectures:

• batch size: 10, 16, 32, 64.

• learning rate: 1e-5, 3e-5, 5e-5.

Training based on the experimental grid resulted in 72 fine-tuned models, 12 for each architecture. Pretrained models were downloaded from “Hugging Face” (Supplementary Table S3). We coded Python scripts (version 3.11.3) for the fine-tuning, testing, and inference using the specialized framework for deep learning PyTorch Lightning (https://github.com/laigen-unam/BERT-trn-extraction). A list of specialized libraries employed in our study is detailed in Supplementary Table S4.

The performance of the 72 fine-tuned models was compared to select the best architecture and the best hyperparameters. The final best model was obtained by fine-tuning the best architecture using the best hyper-parameters with the total sentences from the train and the dev datasets together. We evaluated this final model using the test (held-out) dataset calculating Precision, Recall (Sensitivity), and F1-Score Macro. For these three metrics, 0 is the worst value and 1 is the best value [39]. For a binary classification problem with “positive” and “negative” categories, the Precision score is the fraction of examples predicted correctly by the model:


(2) $$Precision = \,\frac{{TP}}{{TP + FP}},$$

where $TP$ (true positives) are the examples predicted as “positive” category that are actually true; and $FP$ (false positives) are the examples predicted as “positive” category that actually correspond to the “negative” category. The Recall is the fraction of correctly predicted examples of a category among the total examples of that category:


(3) $$Recall = \,\frac{{TP}}{{TP + FN}},$$

where $FN$ (false negatives) are the examples incorrectly predicted as the “negative” category. The F1-Score is the harmonic mean of Precision and Recall:


(4) $$F1 - Score = \,2\frac{{Precision \times Recall}}{{Precision + Recall}}.$$

We used F1-Score Macro, which calculates the statistics for each label and averages them, attributing equal importance to all classes despite the imbalance of the categories. We also calculated the Matthew’s correlation coefficient (MCC), which is recommended for classification problems with imbalanced categories, as it gives a high score only if the classifier correctly predicted most of the categories [40]. The MCC measures the correlation of the true categories *c* with the predicted categories *l*, the worst value is −1, and the best value is 1.


(5) $$\begin{aligned}MCC &= \frac{{Cov\left( {c,l} \right)}}{{{\sigma _c}{\sigma _l}}} \nonumber \\ &= \frac{{\left( {TP \times TN} \right) - \left( {FP \times FN} \right)}}{{\sqrt {\left( {TP + FP} \right) \times \left( {TP + FN} \right) \times \left( {TN + FP} \right) \times \left( {TN + FN} \right)} }},\end{aligned}$$

where $Cov\left(c,l\right)$ is the covariance of the true categories $c$ and the predicted categories $l$; ${\sigma _c}$ and ${\sigma _l}$ are the standard deviations, respectively. The $TN$ (true negatives) are the correctly predicted examples of the negative category.

To expand the understanding of the predictive capabilities of the model, we examined the confusion matrix, the metrics for each category, and the correct and incorrect predictions in the test dataset. A confusion matrix is relevant to observe the performance of a classifier for a multiclass task, because it shows for each pair of classes $\langle c1,c2\rangle $ how many examples of category $c1$ are incorrectly classified as category $c2$, and vice versa. This matrix allows to point out opportunities to improve the performance of a classifier [39], as we may find a more confusing category:


(6) $$Confusion\,matrix = \left( {\begin{array}{*{20}{c}}
{TP}&{FN}\\
{FP}&{TN}
\end{array}} \right).$$

### Salmonella TRN extraction

The curation team of RegulonDB also compiled a set of 3005 sentences containing regulatory interactions of *Salmonella* from 264 biomedical articles employing the same assisted-curation strategy [29]. This dataset was also provided for our study by the RegulonDB curation team. *Salmonella* is one of the main pathogens that infect both humans and animals worldwide [41]. Transcriptional regulation of this bacterium has been studied for a while to face relevant problems, such as antimicrobial resistance [13]. The publication of a TRN for *Salmonella* has also received attention [10].

We utilized this dataset to evaluate the performance of our best model for extracting a TRN using complete articles. This dataset had the same limitation as the dataset of *E. coli* for fine-tuning: sentences did not have marks for the pair of entity mentions that participated in the curated interaction. This dataset only had the three categories of regulation (activator, repressor, and regulator) and lacked the “no_relation” category.

**Figure 3.**
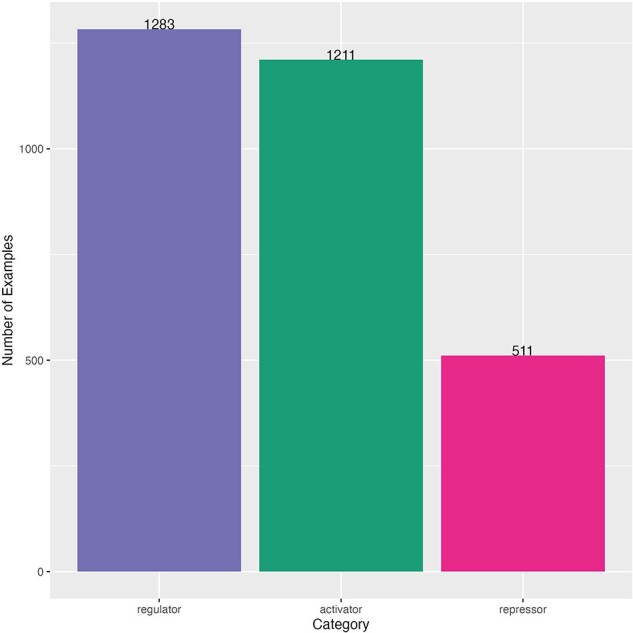
Distribution of curated sentences by category of the dataset for TRN extraction. Sentences from literature of *Salmonella*.

This dataset also had an imbalanced distribution of sentences by category, but the majority category was regulator (1283 sentences), and the minority category was repressor (511 sentences) (Fig. 3). The minimum length of sentence (number of characters) was 23 characters and the maximum length was 2070 characters (mean of 201 and median of 176) (Supplementary Fig. S3). Sentences come from 264 different scientific articles (Supplementary Fig. S4). A comparison of this dataset and the one used for fine-tuning is shown in Supplementary Table S2.

We applied our best model to extract a TRN of *Salmonella* using the 264 complete articles. The idea was to explore the performance of the model to deal with the large-scale extraction of TRNs of different bacteria, which is one of our mid-term goals. For evaluation, we used the dataset with the 3005 sentences curated from the 264 articles; however, instead of measuring the classification of theses sentences, we measured the capability of the model to extract the same regulatory interactions (TF, regulated element, and category) curated from the 264 articles. In other words, we compared the regulatory interactions extracted by the model with the regulatory interactions extracted by the curator. This change in evaluation strategy is mainly because to evaluate our method in the future with a published TRN (e.g. SalmoNet and RegulonDB) we will not have the sentences from which the interactions were curated, but only the set of interactions.

Then, we collected the 264 PDF files. For articles without open-access rights, downloading rights were granted by our institution. PDF files were converted into raw text using an in-house developed tool. Sentence split and tokenization were performed with the Stanford CoreNLP tool [42]. Then sentences were input to the best model to predict the category (classification). The classified sentences were filtered to discard those predicted with the “no_relation” category. From the filtered sentences, we obtained the unique regulatory interactions, that is, a unique combination of a TF, a regulated element and a category. These unique regulatory interactions were searched in the curated dataset to assess the performance of the model. We calculated Precision, Recall, and F1-Score. The metrics were calculated in the same way as for the evaluation of the best model with the test dataset; however, in this calculation, the true positives (*TP*) were the extracted regulatory interactions that were present in the curated dataset; the false positives (*FP*) were the extracted regulatory interactions that were not present in the curated dataset; and the false negatives (*FN*) were the regulatory interactions from the curated dataset that were not extracted by the model [39].

The MCC was not calculated, as we did not have negative examples (false regulatory interactions) in the curated dataset and the number of true negatives could not be obtained. A negative example had to be a sentence mentioning a TF and a regulated element without any transcriptional regulatory relation between them; therefore, we would have had to curate a significant number of sentences to obtain a set of true negative samples. Instead, we focused our efforts on elucidating the capacity of our model to discover TP examples as this is the real situation to evaluate the interactions extracted by our model with a published database. For example, SalmoNet and RegulonDB publish only TP samples of regulatory interactions. The same situation will be expected for all curated databases as the curation work only recovers the TP samples. To get a better understanding of the model performance to extract the TRN, we manually examined a sample of 158 sentences associated with 60 regulatory interactions considered incorrect predictions (FP cases).

## Results and discussion

### Accurate extraction and classification of transcriptional regulatory interactions

In this section, we present the results of the model fine-tuning. Using the early stopping strategy when the cross-entropy loss in the dev dataset did not improve in two epochs, we found a best model (LUKE architecture), which obtained cross entropy of 0.4024 and F1-Score Macro of 0.9107 measured on the dev dataset for fine-tuning. The summary of the hyperparameters found for the best model of each architecture, as well as the metrics, are shown in Table 3. We consider that the high performance of LUKE is due to its architecture being based on the optimization of RoBERTa and that in the pretraining step the entities are independently processed by an entity-aware self-attention mechanism. This capability to represent entities distinctly from the rest of the words was relevant in our classification task to detect regulatory interactions.

**Table 3.** The best hyperparameters for each architecture obtained with the dev dataset for fine-tuning. Sorted by F1-Score. Best result in boldface

Model	F1-Score	Cross entropy	Epoch	Steps	Batch size	Learning rate
**LUKE**	**0.9107**	**0.4024**	**12**	**415**	**32**	**0.00001**
BioBERT	0.8983	0.4633	6	111	64	0.00005
BioLinkBERT	0.8893	0.4137	7	503	16	0.00001
BioRoBERTa	0.8710	0.4547	10	351	32	0.00001
BERT	0.8552	0.5319	7	503	16	0.00003
BioMegatron	0.8434	0.8096	6	440	16	0.00005

**Figure 4.**
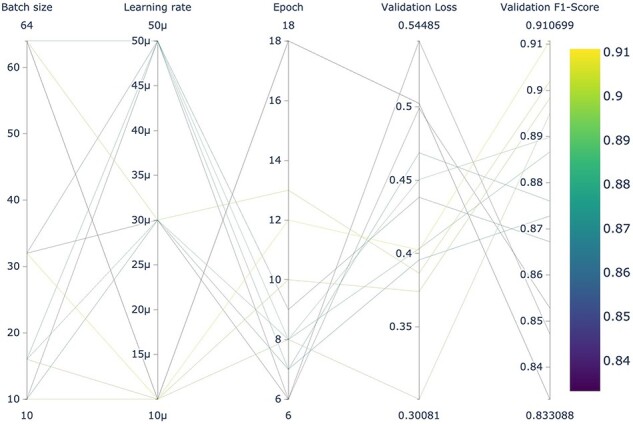
Hyperparameter search of the 12 LUKE models using the dev dataset for fine-tuning.

It is noteworthy that LUKE outperformed specialized transformers pretrained with biomedical literature (BioBERT, BioLinkBERT, BioMegatron, and BioRoBERTa). This may indicate that it is as important to pretrain a BERT-based model using a domain-specific corpus as it is to pretrain the model for a specific task. As far as we know, LUKE had not been previously evaluated for biomedical relation extraction. A graphic summary of the results obtained by the 12 LUKE training runs can be seen in Fig. 4. Figures of remaining architectures are shown in Supplementary Figs. S5–S9. These figures were obtained using “Weights & Biases,” a platform designed for the organization and development of artificial intelligence workflows.

Our best model, based on LUKE architecture, obtained a balanced performance of classification in the test dataset observed in the F1-Score Macro of 0.8685 (Table 4). The Precision score (0.8601) shows that, on average, when the model predicts a category, 86% of the times the prediction is correct. The Recall score (0.8788) indicates that, on average, the model correctly classifies 87% of all input sentences. According to the MCC (0.8163), our model has a relevant performance despite the imbalance of sentences in the categories; this score indicates that our model shows a strong positive correlation between its predictions and the curated categories of the sentences. The decrease in F1-Score obtained with the dev dataset (0.9107) compared to the one obtained in the test (0.8685) shows that the model was over-fitted, a common result for complex deep learning architectures such as transformers. Cross-entropy loss (0.4030) was very similar to the one obtained with the dev dataset.

**Table 4.** Classification report describing the performance of the best model using the test dataset. The two best results for categories in boldface. Global results in italics. Support stands for the number of examples of each category

	Precision	Recall	F1-Score	Support
**Activator**	**0.8965**	**0.9203**	**0.9082**	**113**
No_relation	0.8155	0.8400	0.8275	100
Repressor	0.8545	0.8392	0.8468	56
**Regulator**	**0.9487**	**0.8409**	**0.8915**	**44**
*Macro avg*	*0.8788*	*0.8601*	*0.8685*	*313*
Weighted avg	0.8704	0.8690	0.8691	313

From the analysis of the performance for individual categories (Table 4), we observed that the best classified category was “activator” (F1-Score Macro: 0.9082, support = 113 examples). In addition, this was the top recovered category (Recall: 0.9203) and the second best in Precision (0.8915). It is interesting that the second-best predicted category was “regulator,” although it was also the minority category in the test dataset (support = 44 examples); this fact confirms the capacity of our model to deal with imbalanced categories. Furthermore, the “regulator” category was the class with the highest precision (0.9487, support = 44 examples), showing that our model is reliable when predicting this category. In general, all the categories obtained a relevant F1-Score (above 0.82) regardless of the number of examples (support) of each category.

**Figure 5.**
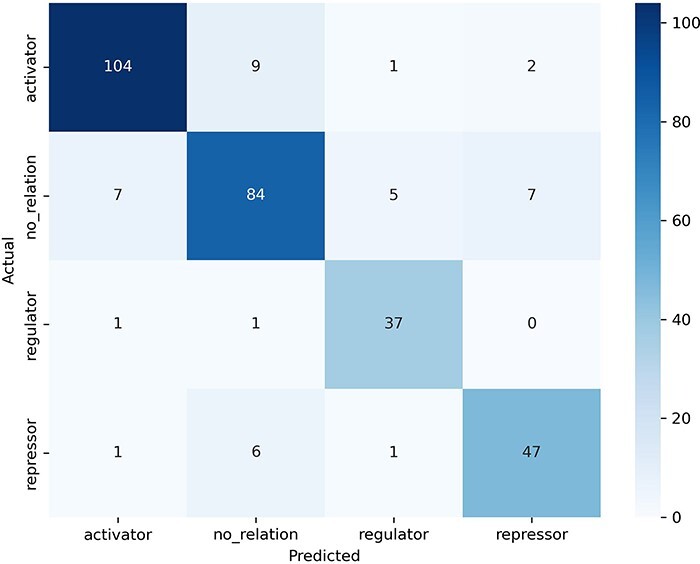
Confusion matrix obtained by the best model in the test dataset.

The examination of the confusion matrix (Fig. 5) allows us to observe which categories our model confuses the most and which the least. The “no_relation” category (support = 100 examples) was the one that caused the most confusion to the model: seven sentences were misclassified as “activator,” five as “regulator,” and seven as “repressor” (second row in the confusion matrix). In turn, predicting the “no_relation” category caused the greatest number of misclassified sentences (16 errors, second column in confusion matrix) despite this being the second category with more examples.

An examination of correct and incorrect classified sentences in the test dataset was performed to extend the understanding of the predictive capabilities of our model. We confirmed that our best model learned different ways to express the regulatory effect. Our model systematically predicts the correct “activator” category for sentences expressing the interaction with the verb “activate” or some morphological variations, such as “activated” and “activation.” The same was observed for the “repress” and “regulate” verbs. Moreover, the model learned different verbs and lexical items expressing the regulatory effect of activation, for example, “enhance,” “stimulate,” “increase,” and induction (rows 1–4 in Supplementary Table S5). The same pattern was observed for the *repressor* category, as the model learned to associate lexical items or phrases to this category, for example, “inhibit” or “negative regulated” (rows 5–6 in Supplementary Table S5). For the “regulator” category, we found that a sentence with the verb “coregulate” was correctly classified (row 7 in Supplementary Table S5).

Regarding the misclassified sentences, we observed that our model correctly predicted some curation errors. For instance, the following sentence was curated as “activator”, but the model predicted the correct category “no_relation,” as the combination of mentions of the GntR TF and the *gntV* gene does not express the regulatory interaction (mentions of entities in boldface); the interaction is rather expressed by the combination of the mention of GntR and the second mention of *gntV* (underlined entities).

- “This and the finding that expression of ***gntV***—lacZ fusion was relatively high even in the absence of cAMP (Table 4) may suggest that binding of **GntR** to all 3 sites slightly activates *gntV* expression.” [43].

Also, classification errors produced by the model were observed. For example, for the following sentence, the curated category was “activator” as the expression of *araC* is activated (“stimulated”) by CAP; however, the model predicted the category “repressor.”

- “The expression of araC is repressed by its own product and stimulated by the CAP system.” [44].

We hypothesize that the verb compound “is repressed by” was more weighted for the model prediction. This hypothesis will be in-depth explored in future work using techniques for transformer interpretability, such as those proposed by ref. [45]. In addition, this sentence shows an interesting case, as this unveils another way to express regulatory interactions: “autoregulation”, which is expressed with the phrase “is repressed by its own product.” This type of regulatory interactions cannot be extracted using our approach of passing to the model the mentions of entities from a predefined list. An option to address this issue is to mark “its own product” being the regulatory entity.

Another case is shown in the following sentence, where the model predicted the category “activator” for the interaction between NarL and *dmsA*, but the curated category was “repressor.”

- “Similarly, at the FNR—activated NarL—repressed dmsA promoter NarL protects a large region that includes the sites for both FNR and RNA polymerase binding.” [46].

We consider that the model prediction was due to the presence of the word “activated,” which may be more weighted for the model than the word “repressed” (again, a future study of interpretability is required). Nevertheless, the expression of the interaction is complex, as the sentence indeed has two nested regulatory interactions written with the TF and the regulatory effect expressed as a past participle: “FNR - activated NarL - repressed dmsA.” This sentence expresses, in a complex compressed form, that *dmsA* is repressed by NarL and it is activated by FNR. More examples of classification errors are available in Supplementary Table S5.

### Most of the Salmonella TRN was accurately extracted

To show an application of our approach, we used our best model to extract a TRN from 264 complete articles of transcriptional regulation of *Salmonella*. As our best model was of LUKE architecture, we had to obtain the span of the entity mentions within the sentences. Then, we automatically obtained the spans of the pair of entity mentions by searching the entities within each sentence of the 264 articles. From the total sentences of the articles, 14 349 had the combination of at least one TF and one regulated element. Then, we classified with our best model the 14 349 sentences. From the classified sentences, we discarded those with “no_relation” category, obtaining 8256 sentences. As we want to extract a TRN from those sentences, we obtained unique regulatory interactions, that is, a unique combination of TF, regulated element and category. Then, 1826 unique interactions were obtained, including 90 unique TFs and 324 unique regulated elements.

The regulatory interactions of the *Salmonella* TRN were compared with the regulatory interactions manually extracted from the same articles by the curation team of RegulonDB. Therefore, we obtained the unique regulatory interactions from the curated dataset (section 2.4). We identified 909 unique curated regulatory interactions, which contained 91 unique TFs and 348 unique regulated elements. We observed a very similar distribution of interactions between curated and predicted categories (Supplementary Table S6).

We summarize the results and evaluation of the TRN extraction in Table 5. We found that our best model was able to correctly extract and determine the type of regulation for 82% of the network (Recall: 0.8217) (Table 5). Nevertheless, our model extracted the double of curated interactions, which results in a low Precision score (0.4090) due to the high number of apparently FP (extracted interactions that did not appear in curated interactions). Evidently, these FPs include incorrect predictions, but note that in fact many of these might well be correctly predicted interactions absent in the curated dataset. To review this aspect, we manually checked 60 randomly selected FP interactions. Note that one interaction may be extracted from several sentences; therefore, we reviewed the 158 sentences of the 60 selected FP interactions.

**Table 5.** Summary of the results and evaluation of *Salmonella* TRN extraction with our best model

Curated interactions	909
Extracted interactions	1826
True positives	747
False negative	162
False positive	1079
Precision	0.4090
Recall	0.8217
F1-Score	0.5462

We found that 60% of reviewed cases were misclassified sentences and the remaining 40% of cases were correctly predicted by the model, corresponding to 25 new regulatory interactions that were not recovered by the curation team (Supplementary Table S7). This finding demonstrates that our model may be beneficial to the curation work. To deal with the FP in future work, we may consider some strategies:

- Joining the curated datasets of *E. coli* and *Salmonella* to increase examples for fine-tuning the BERT-based models.
- Using the probability calculated by the model for the predicted categories to extract the most confident ones.

The performance of our model extracting the majority of the curated *Salmonella* TRN shows that we may assist the manual curation of TRNs of diverse bacteria. However, a large-scale evaluation using complete networks published in databases, such as RegulonDB [9] or SalmoNet [10], is required, being an attractive opportunity to improve the method.

### Limitations of the approach

A limitation of our approach is that we would require a list (dictionary) of TFs and regulated elements to extract TRNs of different bacteria. We were able to perform the anonymization process, because our datasets included the entities participating in the interactions. In other words, to train our model, our datasets included: TF, gene, and sentence; therefore, we anonymized the entities in the sentence with the predefined tags @TF$ and @Regulated$. Nevertheless, to apply our model in the future for a different bacterium, our approach will require a list of entities to be anonymized in the sentences of that bacterium, which may be a problem. Another option if we cannot obtain a list of entities is to automatically recognize those entities using an automatic procedure for biomedical entity recognition (genes, chemicals, drugs, and diseases), which is called in NLP as “Biomedical Named Entity Recognition” (NER). This procedure will allow us to recognize the TFs and genes in sentences of different bacteria, so we will be able to anonymize those entities in the sentences and use our model. Nevertheless, the development of strategies for biomedical NER is an active field [47], so we consider that the selection, evaluation and implementation of a NER system will not be effortless. A simpler strategy will be to start by generating a first version of those dictionaries from specialized biological databases like GenBank or BioCyc.

Another limitation is that the initial dataset curated by the team of RegulonDB included only sentences with correct regulatory interactions. This fact may be applicable to all datasets created by curation, as curation work implies recovering what is true or correct. It is possible that our model has learned more patterns associated with correct interactions than patterns associated with dubious interactions (negation, speculation, and lack of certainty), despite the use of the “no_relation” category. For example, the following sentence expresses a lack of regulation between YdiV and *flhDC*, but these kinds of sentences were not recovered in curation works. Our model incorrectly classified this sentence as “regulator.”

- “These average MFIs did not differ significantly between the strains (Fig. 3B), indicating that **YdiV** does not regulate ***flhDC*** transcription.” [48].

Detecting negation, speculation, certainty, and other nuances to express interactions (called “meta-knowledge dimensions”) has been in the interest of the biomedical relation extraction field for a while [49]. In future work, we will explore recent studies, especially those based on pretrained transformer models, to detect these dimensions to improve our extraction [50, 51].

## Conclusions

In this study, a BERT-based model with significant performance to classify and extract transcriptional regulatory interactions of bacteria from biomedical literature was obtained. To the best of our knowledge, this study is the first to extract interactions between TFs and genes/operons using BERT-based approaches. The automatic or assisted extraction of regulatory interactions is of high relevance given the rich amount of knowledge present in the literature awaiting its extraction and incorporation in databases. Our approach extracted most of the curated TRN of *Salmonella* using complete articles. The examination of model predictions confirmed that our approach may extract interactions absent in the curated network. We consider our work as a relevant starting point to address the limitations of access to biomedical knowledge, especially for the extraction of TRNs of different bacteria and diseases of biological interest.

## Supplementary Material

baae094_Supp

## Data Availability

The datasets supporting the conclusions of this article are available in the GitHub repository [https://github.com/laigen-unam/BERT-trn-extraction].
