# Supplementary material for: Automatic extraction of transcriptional regulatory interactions of bacteria from biomedical literature using a BERT-based approach
Source: Database (Oxford). 2024 Aug 30;2024:baae094. doi: 10.1093/database/baae094 (PMC11363960; doi:10.1093/database/baae094)
Supplement: baae094_Supp [file baae094_supp.zip › suppl_data/Supplementary_Material.pdf]

Automatic extraction of transcriptional regulatory interactions of bacteria from biomedical literature using a BERT-based approach

Supplementary material

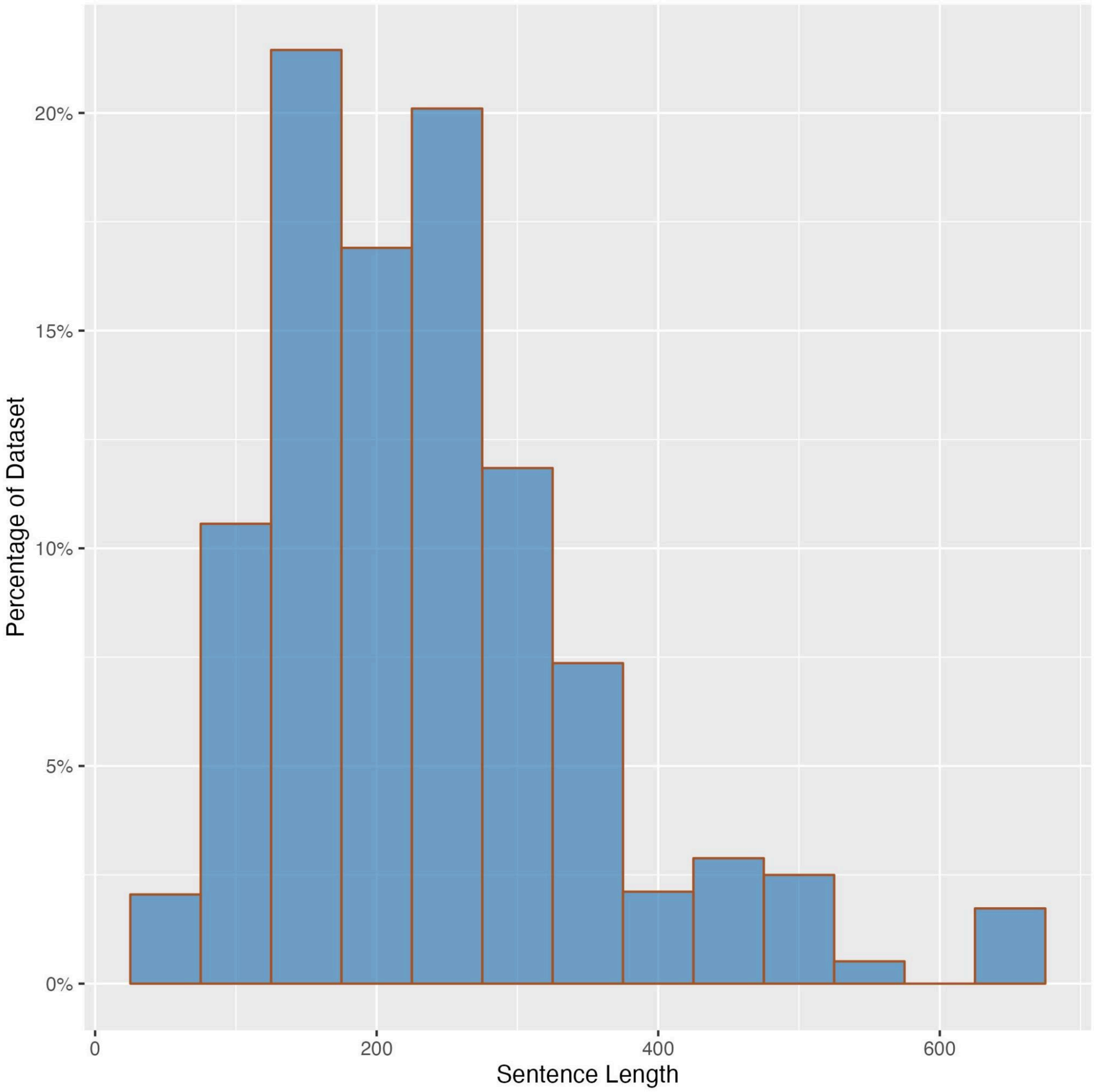

Figure S1. Distribution of sentence length (number of characters) in the dataset for fine-tuning.

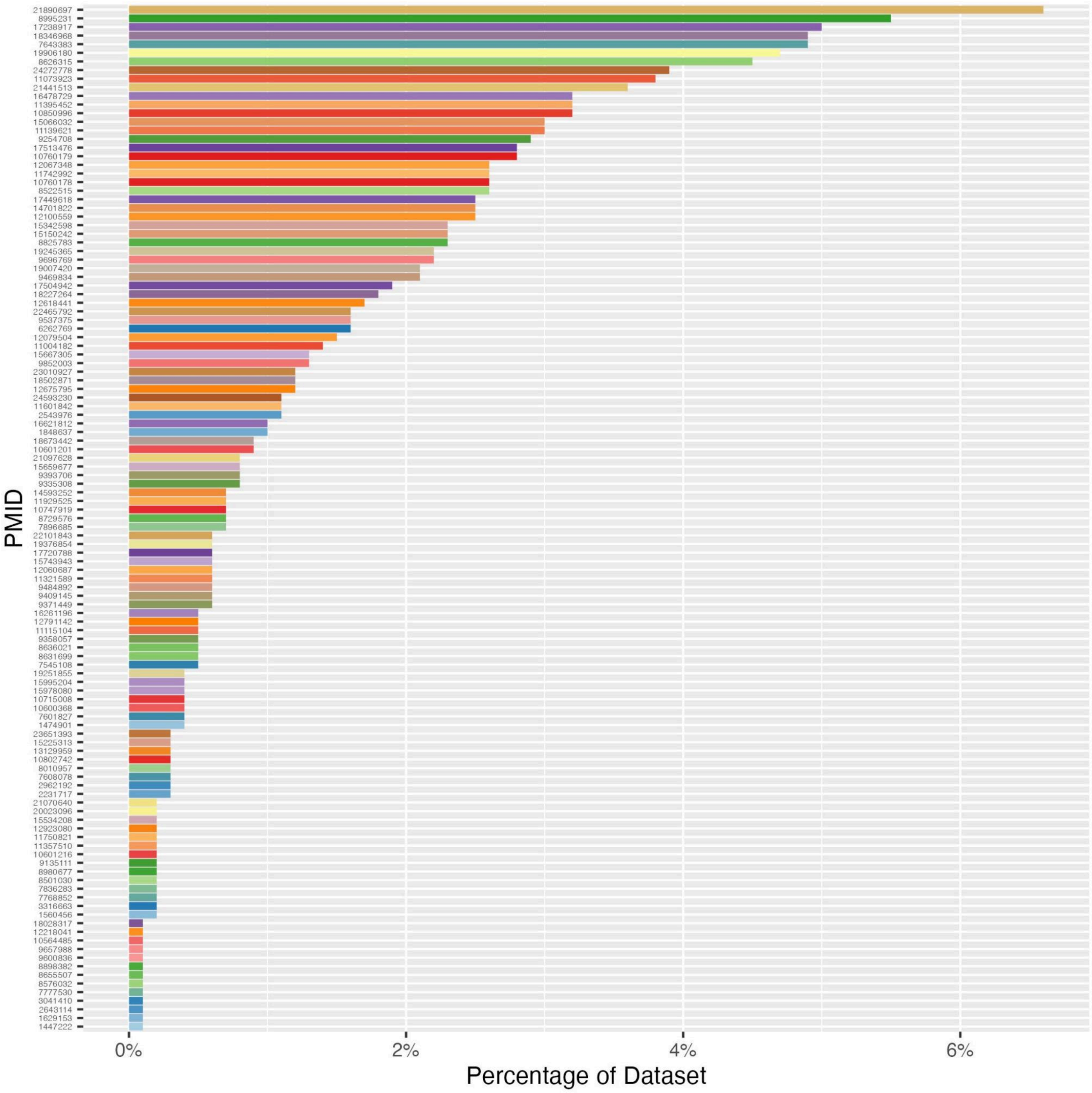

Figure S2. Percentage of sentences per publication (PMID) in the dataset for fine-tuning.

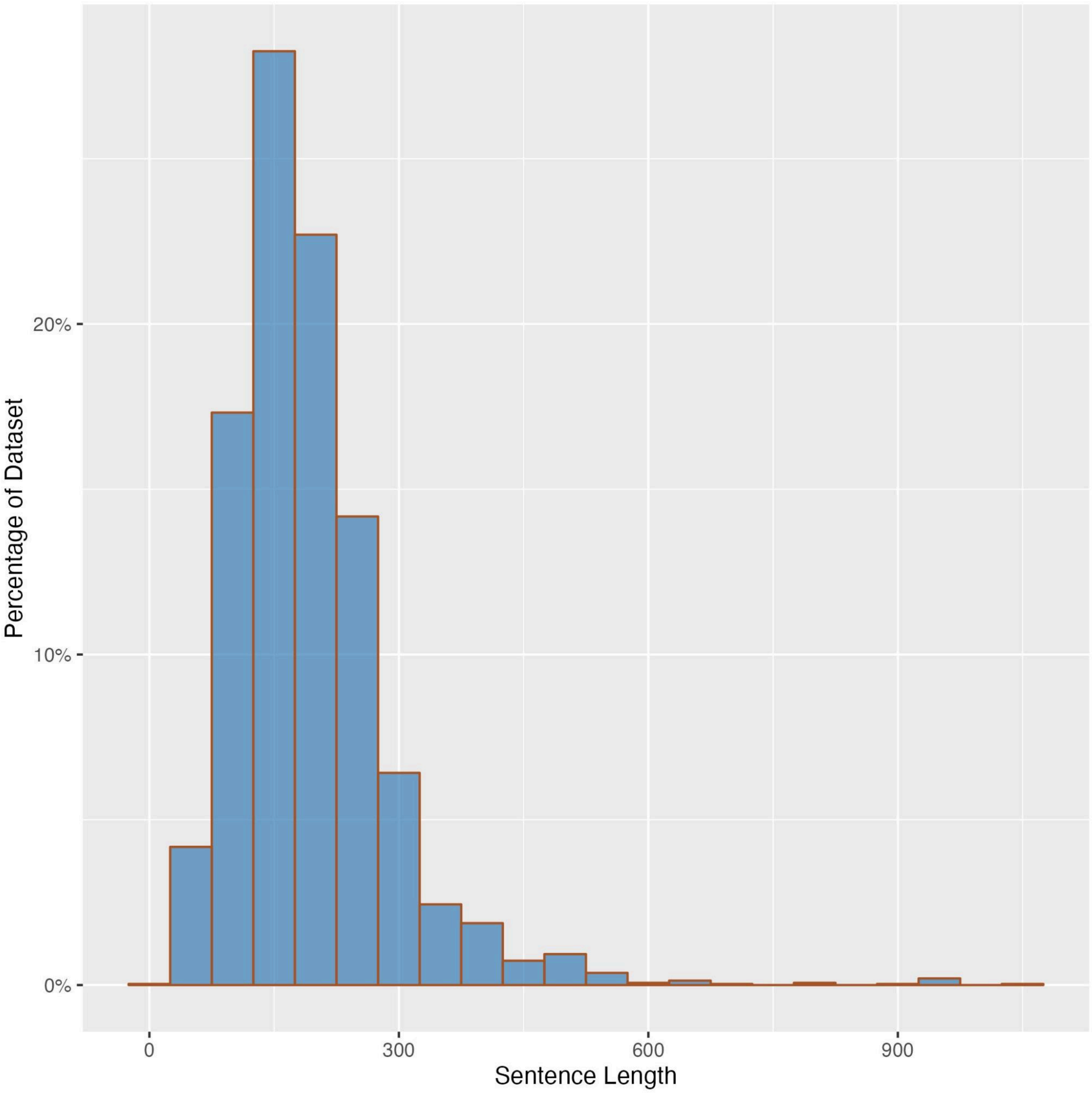

Figure S3. Distribution of sentence length (number of characters) in the dataset for *Salmonella* TRN extraction.

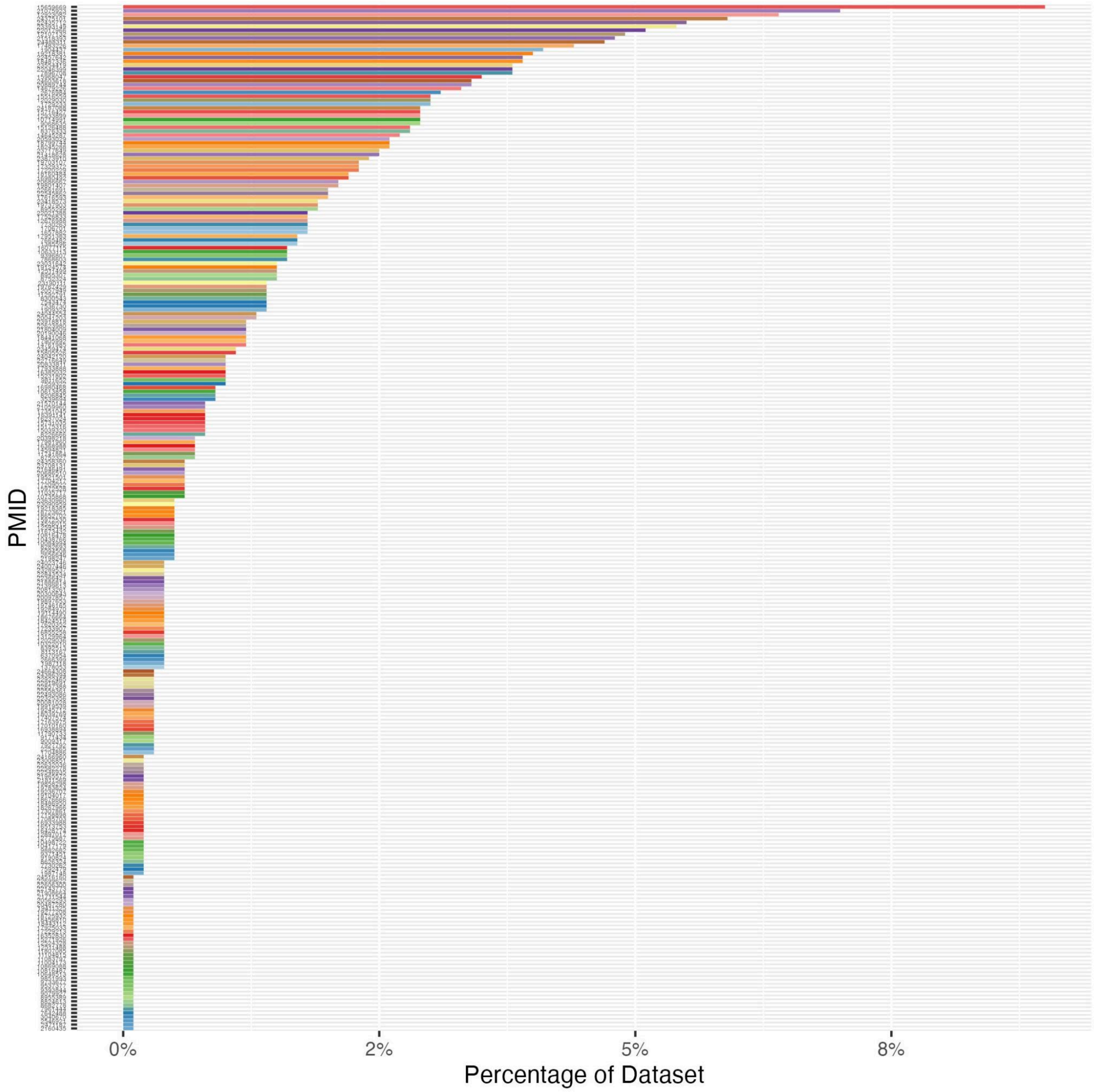

Figure S4. Percentage of sentences per publication (PMID) in the dataset for *Salmonella* TRN extraction.

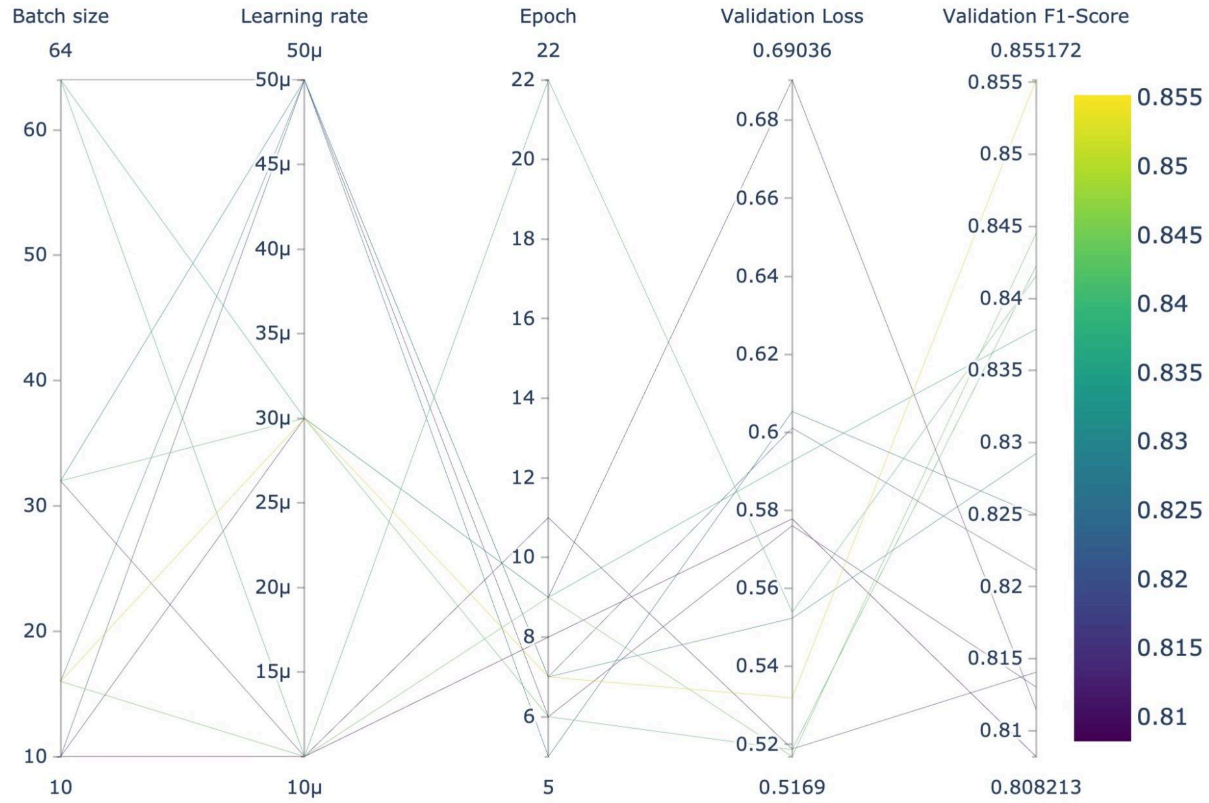

Figure S5. Hyper-parameter search of the twelve BERT models using the dev dataset for fine-tuning.

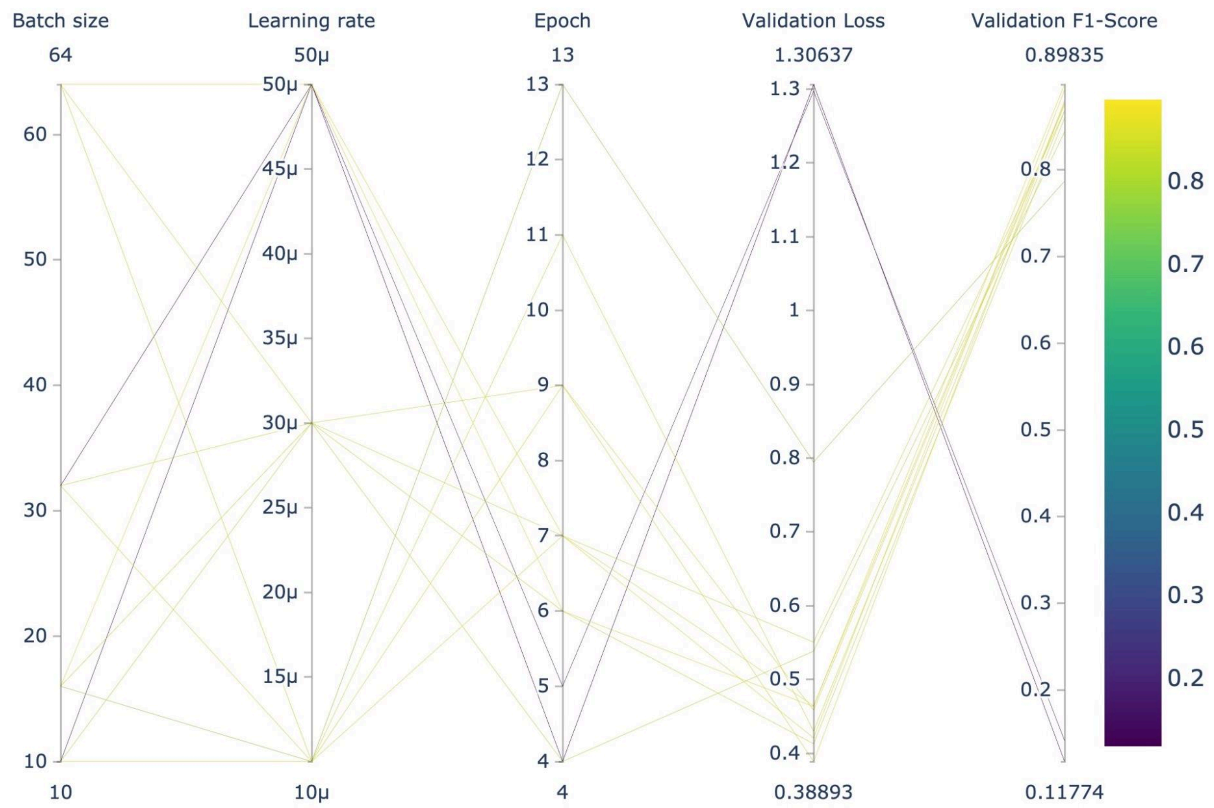

Figure S6. Hyper-parameter search of the twelve BioBERT models using the dev dataset for fine-tuning.

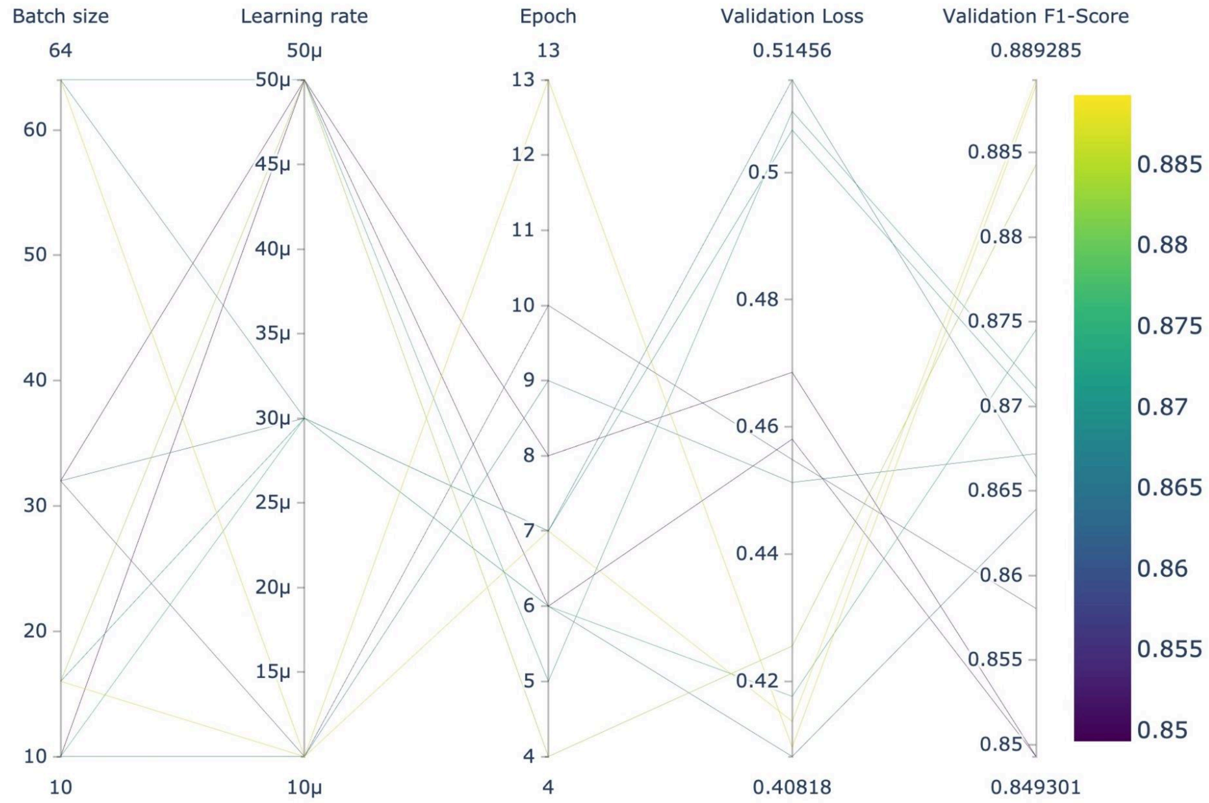

Figure S7. Hyper-parameter search of the twelve BioLinkBERT models using the dev dataset for fine-tuning.

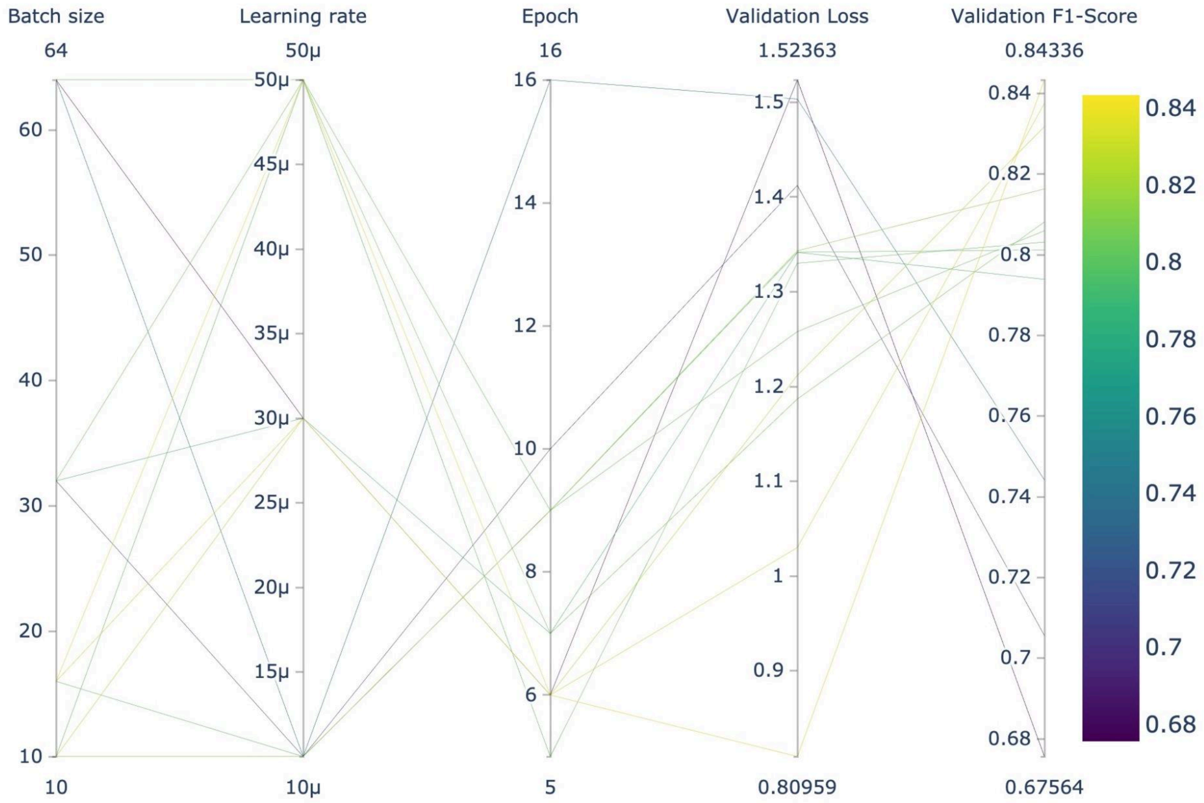

Figure S8. Hyper-parameter search of the twelve BioMegatron models using the dev dataset for fine-tuning.

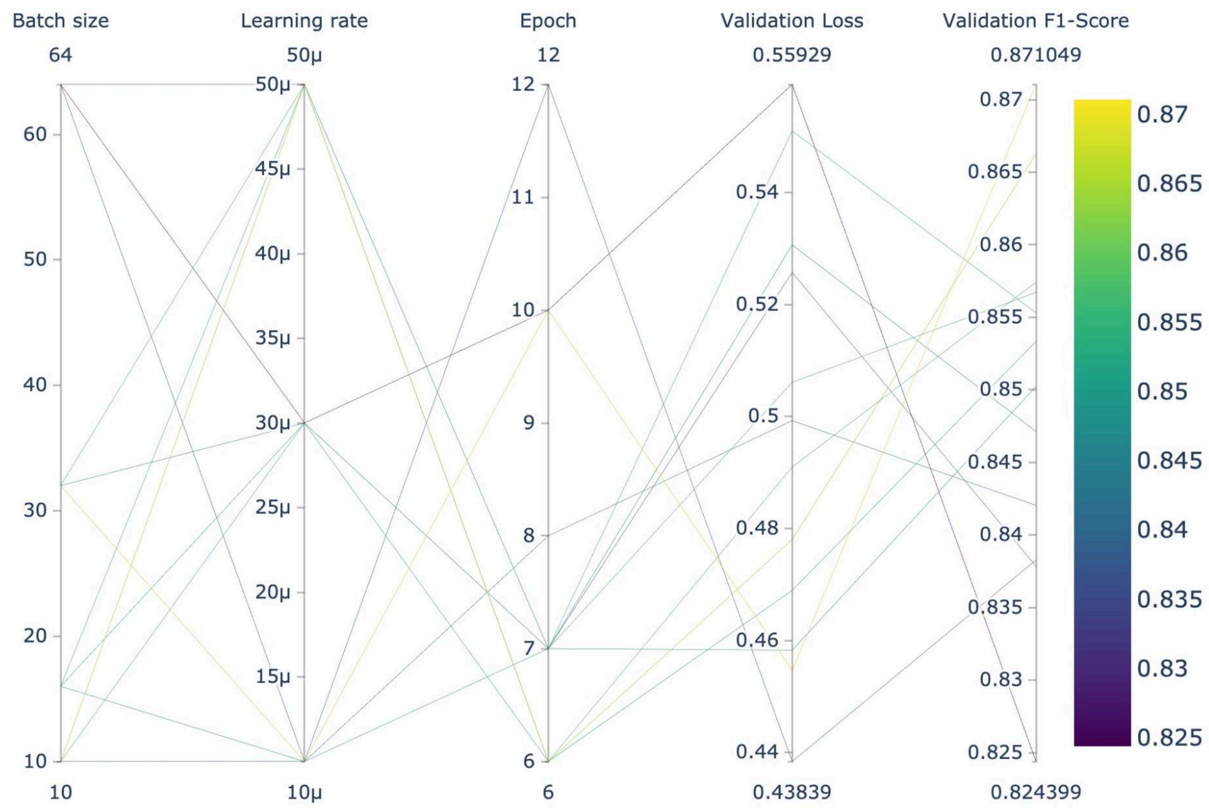

Figure S9. Hyper-parameter search of the twelve BioRoBERTa models using the dev dataset for fine-tuning.

Table S1. Distribution of sentences in the complete dataset and train, dev and test datasets. Number of sentences followed by their percentage.

**Category**	**Complete**	**Train**	**Dev**	**Test**
activator	593 (38%)	381 (38%)	99 (40%)	113 (36%)
no_relation	493 (32%)	316 (32%)	77 (31%)	100 (32%)
repressor	269 (17%)	169 (17%)	44 (18%)	56 (18%)
regulator	207 (13%)	133 (13%)	30 (12%)	44 (14%)
	1562 (100%)	999 (100%)	250 (100%)	313 (100%)

Table S2. Comparison of characteristics between the dataset for fine-tuning (*E. coli*) and the dataset to extract the *Salmonella* TRN (model application).

Characteristic	Dataset for fine-tuning	Dataset for model application
Number of sentences	1562	3005
% examples of Activator category	38%	40%
% examples of Repressor category	17%	17%
% examples of Regulator category	13%	43%
Number of transcription factors	66	91
Number of regulated elements	200	348
Median of sentence length	223	176

Table S3. Pre-trained BERT models downloader for our study.

Model	Link for the implementation
BERT	https://huggingface.co/bert-base-uncased
BioBERT	https://huggingface.co/dmis-lab/biobert-v1.1
BioLinkBERT	https://huggingface.co/michiyasunaga/BioLinkBERT-base
BioMegatron	https://huggingface.co/EMBO/BioMegatron345mUncased
BioRoBERTa	https://huggingface.co/allenai/biomed_roberta_base
LUKE	https://huggingface.co/studio-ousia/luke-base

Table S4. Main specialized libraries and tools employed in our study.

Library	Version	Description
pandas	1.5.2	Data analysis and manipulation
seaborn	0.12.2	Data visualization

matplotlib	3.6.2	Data visualization
scikit-learn	1.0.2	Data split and classification report
torch	2.0.1	Loading sentences to model by mini-batches and cross entropy
pytorch-lightning	2.0.6	Building deep learning models
transformers	4.29.2	Tokenizer, transformer models with softmax classification layer and AdamW optimizer
wandb	0.15.8	Tracking training metrics in real time and sweeps for hyperparameter search
torchmetrics	1.0.3	Confusion matrix and Multiclass metrics: Precision, Recall, F1-Score, Matthew's Correlation Coefficient

Table S5. A sample of correct and incorrect classified sentences in the test dataset. Pair of mentions of entities are in boldface. Sentences are tokenized, so words, punctuation, numbers, and symbols are separated by spaces.

#	Sentence	Correct category	Predicted category
1	*The ArgP protein enhances the expression of the argK gene ArgP argK (Celis, 1999).*	activator	activator
2	*Lrp stimulates transcription of lysP by direct binding to its control region (Ruiz et al., 2011).*	activator	activator
3	*AraC - dependent transcription initiation at the araBAD promoter is increased by CRP (Howard et al., 2002).*	activator	activator
4	*MelR is essential for induction of the melAB operon that is responsible for melibiose metabolism . (Wade et al., 2000).*	activator	activator
5	*Thus , it appears that NarL and NarP adopt overlapping mechanisms to inhibit ydhY – T expression . (Partridge et al., 2008).*	repressor	repressor
6	*The treB treC operon is negatively regulated by TreR , whose gene treR is located upstream of treB but is not part of the operon . (Klein et al., 1995)*	repressor	repressor
7	*Expression of acrZ is coregulated with acrAB and tolC by the MarA , Rob , and SoxS transcription factors .(Hobbs et al., 2012).*	regulator	regulator
8	*Both the aerobic and anaerobic expression levels of dcuB were only ca . twofold lower in the arcA mutant (JRG3841) , indicating that **ArcA** plays no more than a minor role in regulating **dcuB** expression in response to oxygen (Fig . 5B) and that ArcA is not responsible for the FNR - independent mechanism of anaerobic activation of dcuB transcription .* (PMID 9852003)	activator	no_relation
9	*This shows that we had failed to identify MelR mutants with improved specificity for the changed KK433 **MelR** - binding sequences ; mutants with specificity for the KK433 sequence would have given lower levels of activation with the wild - type **melAB** promoter .* (PMID 8010957)	activator	no_relation

10	***marA*** *expression is repressed by MarR and is derepressed by the interaction of* ***MarR*** *with various phenolic compounds such as salicylate .* (PMID 12067348)	no_relation	repressor
11	*However the much stronger repression of this fusion by overproduced Mlc , compared with overproduced* ***NagC*** *, shows that the isolated* ***nagE*** *operator site has a higher affinity for Mlc than NagC .* (PMID 11139621)	no_relation	repressor
12	*Thus , the* ***melR*** *promoter is not efficiently repressed by* ***MelR*** *, and MelR is over - expressed . (PMID 18346968)*	no_relation	repressor
13	*It is unlikely that CRP binding to site 4 contributes directly to an increase in* ***rhaSR*** *expression, since transcription activation by* ***CRP*** *requires that its binding site be on the same face of the DNA as the promoter (6).* (PMID 11073923)	no_relation	activator
14	*The location of the* ***AraC*** *binding site upstream of* ***ytfQ*** *is too far upstream of the transcription start site to repress transcription by directly occluding RNAP .* (PMID 24272778)	repressor	regulator
15	***MelR*** *carrying each of the single substitutions is less able to repress the* ***melR*** *promoter , while MelR carrying some combinations of substitutions is completely unable to repress the melR promoter .* (PMID 16621812)	repressor	no_relation
16	*On the other hand , activation of the P* ***araB*** *promoter was delayed when glucose was present , consistent with the regulation of this promoter also by* ***CRP*** *(6) .* (PMID 20023096)	regulator	activator
17	*For instance , Rob has been shown to bind and activate the* ***zwf*** *promoter in vitro but whole cell zwf regulation cannot be activated by Rob , although the gene responds to* ***SoxS*** *and MarA (Ariza et al . , 1995 ; Jair et al . , 1995 1996a ; b).* (PMID 12100559)	regulator	no_relation

Table S6. Comparison of the distribution of interactions in the predicted and curated categories for *Salmonella* TRN extraction.

Category	Number of curated interactions	Number of predicted interactions
Regulator	378 (42%)	710 (39%)
Activator	321 (35%)	661 (36%)
Repressor	210 (23%)	455 (25%)
Total	909 (100%)	1826 (100%)

Table S7. The 25 true *Salmonella* regulatory interactions extracted by our model that were not in curated dataset.

Transcription factor	Regulated element	Predicted category
HU	hilA	activator
PhoB	hilD	regulator

ArcA	rpoS	activator
DeoR	deoQ	repressor
PhoP	orgBC	activator
PmrA	phoP	activator
MviA	igaA	activator
RpoS	hilA	regulator
RcsB	srfJ	activator
Mlc	hilD	regulator
MviA	katE	activator
MviA	iraP	activator
PhoB	hilD	repressor
PreA	pmrCAB	activator
SprB	hilA	repressor
Fis	invE	activator
FlhDC	hilD	repressor
InvF	slrP	activator
RtsA	prgHIJK	repressor
OxyR	metH	regulator
RpoS	bapA	regulator
CRP	putA	activator
PhoP	ompD	regulator
FimW	fimW	regulator
RpoS	cacA	regulator
